# Health and societal effects from exposure to fragranced consumer products

**DOI:** 10.1016/j.pmedr.2016.11.011

**Published:** 2016-11-14

**Authors:** Anne Steinemann

**Affiliations:** Department of Infrastructure Engineering, Melbourne School of Engineering, The University of Melbourne, Melbourne, Victoria 3010, Australia; College of Science, Technology and Engineering, James Cook University, Townsville, Queensland 4811, Australia; Climate, Atmospheric Sciences, and Physical Oceanography, Scripps Institution of Oceanography, University of California, San Diego, La Jolla, CA 92093, USA

**Keywords:** Fragranced consumer products, Migraines, Asthma, Fragrance-free policies, Indoor air quality

## Abstract

Fragranced consumer products—such as air fresheners, cleaning supplies, and personal care products— pervade society. This study investigated the occurrence and types of adverse effects associated with exposure to fragranced products in Australia, and opportunities for prevention. Data were collected in June 2016 using an on-line survey with a representative national sample (*n* = 1098). Overall, 33% of Australians report health problems, such as migraine headaches and asthma attacks, when exposed to fragranced products. Of these health effects, more than half (17.1%) could be considered disabling under the Australian Disability Discrimination Act. Additionally, 7.7% of Australians have lost workdays or a job due to illness from fragranced product exposure in the workplace, 16.4% reported health problems when exposed to air fresheners or deodorizers, 15.3% from being in a room after it was cleaned with scented products, and 16.7% would enter but then leave a business as quickly as possible due to fragranced products. About twice as many respondents would prefer that workplaces, health care facilities and professionals, hotels, and airplanes were fragrance-free rather than fragranced. While 73.7% were not aware that fragranced products, even ones called green and organic, emitted hazardous air pollutants, 56.3% would not continue to use a product if they knew it did. This is the first study in Australia to assess the extent of adverse effects associated with exposure to common fragranced products. It provides compelling evidence for the importance and value of reducing fragranced product exposure in order to reduce and prevent adverse health effects and costs.

## Highlights

• One-third of the Australian population report adverse health effects from fragranced products.
• Common effects include respiratory problems, migraine headaches, and asthma attacks.
• Fragranced products resulted in lost workdays and jobs, and restricted access in society.
• About twice as many people would prefer fragrance-free rather than fragranced indoor environments
• Over 70% were not aware that fragranced products emitted hazardous air pollutants.

## 1 Introduction

Contrary to popular belief, most exposure to hazardous pollutants that affect health and well-being occurs indoors (Ott et al., 2007, Brown, 2007). A primary source of these indoor pollutants and exposures is common fragranced consumer products, such as air fresheners, cleaning products, laundry supplies, and personal care products (Cheng et al., 2015, Nazaroff and Weschler, 2004, Steinemann et al., 2011).

Exposure to fragranced products has been associated with a range of adverse human health effects, including migraine headaches, contact dermatitis, asthma attacks, respiratory difficulties, and mucosal symptoms (e.g., Kelman, 2004, Caress and Steinemann, 2009, Elberling et al., 2005, Millqvist et al., 1999, Johansen, 2003, Kumar et al., 1995). In two previous surveys, Caress and Steinemann (2009) found that 17.5% and 20.5% of the general US population (between 2002–3 and 2005–6 respectively) reported breathing difficulties, headaches, or other health problems when exposed to air fresheners and deodorizers.

Fragranced consumer products emit dozens of different volatile compounds, including terpenes (e.g., limonene, alpha-pinene, and beta-pinene) that are primary pollutants, and that react with ozone to generate secondary pollutants such as formaldehyde and acetaldehyde (Nazaroff and Weschler, 2004). Even so-called green and organic fragranced products emit hazardous pollutants, similar to regular fragranced products. Little information exists, however, on potentially hazardous compounds emitted from fragranced products, in part because products are not required to disclose all ingredients (Steinemann, 2015). Thus, knowledge of potential exposures and effects is essential to effective risk reduction.

This study investigates the occurrence and types of exposures to fragranced products and associated health and societal effects in the Australian population. Further, it investigates the potential for preventive measures, such as fragrance-free policies, to reduce health risks and costs.

## 2 Methods

An on-line survey was conducted of the adult Australian population, using a national random sample representative of age, gender, and state (*n* = 1098, 95% confidence level with a 3% margin of error). The survey instrument, a 35-item questionnaire, was developed and tested over a two-year period, including cognitive testing with 10 individuals and piloting with over 100 individuals, before full implementation in June 2016. The survey drew upon participants from a large web-based Australian panel (over 200,000 people) held by Survey Sampling International. Participant recruitment followed a randomized process (SSI, 2016) with an open invitation, rather than a direct invite, to the pool of panelists available at the time. The pool was filtered to achieve a representative sample through a set of initial questions for basic demographic characteristics. All responses were anonymous. Average survey completion time was approximately 10 min. Survey response rate was 93%. Only completed questionnaires were included in the final data analysis. The research study received ethics approval from the University of Melbourne. Details of the survey methodology, as well as statistical analyses of questionnaire data and for results summarized below, are provided as supplemental documents.

The questionnaire investigated both personal and public exposure to fragranced products, health effects related to exposures, impacts of fragrance exposure in the workplace and in public places, awareness of fragranced product ingredients and labeling, preferences for fragrance-free environments and policies, and demographic information. The questionnaire provided one question on each page, with multiple choice and open format answers; five sets of questions were randomized for their multiple choice items, and eight questions were conditionally displayed based on responses to other items. Data were collected and analyzed in June 2016.

Fragranced consumer products were investigated in the following categories: (a) Air fresheners and deodorizers; (b) Personal care products; (c) Cleaning supplies; (d) Laundry products; (e) Household products; (f) Fragrance; and (g) Other. Health effects were investigated in the following categories: (a) Migraine headaches; (b) Asthma attacks; (c) Neurological problems; (d) Respiratory problems; (e) Skin problems; (f) Cognitive problems; (g) Mucosal symptoms; (h) Immune system problems; (i) Gastrointestinal problems; (j) Cardiovascular problems; (k) Musculoskeletal problems; (j) Other health problems. The categories of fragranced products and health effects were developed from prior studies (Steinemann, 2015, Caress and Steinemann, 2009, Miller and Prihoda, 1999), and pre-tested and piloted with over 100 individuals, including health care professionals, before full survey implementation.

## 3 Results

Overall, 98.5% of the Australian population is exposed to fragranced products at least once a week from either their own use (98%), others' use (88.1%), or both. From their own use, 66.8% are exposed to air fresheners and deodorizers at least once a week; 91.6% personal care products; 83.2% cleaning supplies; 84.3% laundry products; 77.1% household products; 69.6% fragrance; 2.3% other. From others' use, 50.8% are exposed to air fresheners and deodorizers at least once a week; 61.5% personal care products; 50.7% cleaning supplies; 44.3% laundry products; 49.6% household products; 67.8% fragrance; 1.8% other.

Importantly, 33% of the general population reported one or more types of health problems associated with exposure to one or more types of fragranced products. The most common types of adverse health effects were as follows: 16.7% of the population reported respiratory problems; 14.0% mucosal symptoms; 10.0% migraine headaches; 9.5% skin problems; 7.6% asthma attacks; 4.5% neurological problems; 4.1% cognitive problems; 3.3% gastrointestinal problems; 3.3% immune system problems; 3.0% cardiovascular problems; 2.6% musculoskeletal problems; and 1.9% other.

When exposed to air fresheners or deodorizers, 16.4% experience health problems; these include respiratory problems (9.1%), mucosal symptoms (6.2%), skin problems (4.8%), asthma attacks (4.5%), migraine headaches (4.2%), neurological problems (2.2%), among other adverse effects. In addition, in other types of exposure situations, 15.3% reported health problems from being in a room after it was cleaned with scented products, 6.1% from the scent of laundry products from dryer vents, and 19.4% from being near someone wearing a fragranced product. For 17.1% of the population, the severity of the health problems was reported to “result in a total or partial loss of bodily or mental functions,” which is a criterion for determining disability under the Australia Disability Discrimination Act (DDA, 1992).

Fragranced products also hindered access in society. Of the general population, 11.6% are unable or reluctant to use the toilets in a public place, because of the presence of an air freshener, deodorizer, or scented product. Also, 10.3% are unable or reluctant to wash their hands with soap in a public place, because they know or suspect that the soap is fragranced. Further, 15.0% have been prevented from going to some place because they would be exposed to a fragranced product that would make them sick. Interestingly, 16.7% of the population reported that if they enter a business, and smell air fresheners or some fragranced product, they want to leave as quickly as possible. Finally, 7.7% have lost work days or a job (in the past 12 months) due to exposures to fragranced products in the workplace.

Fragranced products emit a range of chemicals, including hazardous air pollutants, but ingredients do not need to be fully disclosed on the product label or material safety data sheet. Even so-called green and organic fragranced products can emit hazardous pollutants, similar to regular products (Steinemann, 2015). Of the population surveyed, 47.2% were not aware that a “fragrance” in a product is typically a chemical mixture of several dozen to several hundred chemicals, 68.6% were not aware that fragrance chemicals do not need to be fully disclosed on the product label or material safety data sheet, 68.9% were not aware that fragranced products typically emit hazardous air pollutants such as formaldehyde, and 73.7% were not aware that even so-called natural, green, and organic fragranced products typically emit hazardous air pollutants. However, 56.3% would not still use a fragranced product if they knew it emitted hazardous air pollutants.

Fragrance-free indoor environments received widespread support. Of the general population, 42.8% would be supportive of a fragrance-free policy in the workplace (compared with 22.2% that would not), 43.2% would prefer that health care facilities and health care professionals be fragrance-free (compared with 25.2% that would not). Also, 57.7% would prefer flying on an airplane without scented air pumped through the passenger cabin (compared with 16.3% with scented air), and 55.6% would prefer staying in a hotel without fragranced air (compared with 22.7% with fragranced air).

## 4 Discussion

The problem of fragranced products is sweeping Australia and other countries, resulting in adverse health effects, lost workdays, and inability to access public places, such as restrooms and businesses. While the use of fragranced products may be premised on that they improve indoor air quality, the contrary is actually the case; that is, fragranced products emit and generate a complex mixture of chemical pollutants, including carcinogenic hazardous air pollutants, but nearly all are undisclosed. While further research is needed to better understand which chemicals and mixtures are associated with the effects, what is known is that the products are reportedly causing adverse effects in a sizeable (33%) percentage of the population. Further, the effects can be immediate, severe, and potentially disabling.

Important implications for prevention arise from this study. First, for workplaces and other environments, fragrance-free policies would be a logical step, benefiting employees, employers, and the public. Such policies have been implemented in workplaces, schools, hospitals, and public and private buildings around the world. As an example, the US Centers for Disease Control and Prevention, Indoor Environmental Quality Policy (CDC, 2009) states that “Scented or fragranced products are prohibited at all times in all interior space owned, rented, or leased by CDC.” Second, for individuals, fragranced products can be removed from use, or swapped out for fragrance-free products with similar functionality. A fragrance in a product is not intended to clean the air or reduce air pollutants. Thus, it could be asked whether the perceived benefits of use are dwarfed by the costs to personal and public health. Third, for businesses, fragranced products may actually repel more customers than attract, as well as create potential liability; e.g., the use of air fresheners in a business can cause potentially disabling effects in customers. Fourth, for medical professionals and patients, when faced with health problems such as headaches, respiratory difficulties, mucosal symptoms, rashes, asthma, and others, consider the possibility that fragranced products could be a contributor. Finally, for public officials, the problem of “secondhand scents,” or indirect exposure to fragranced products, has parallels to secondhand tobacco smoke. Prevention from fragrance product exposure will enable individuals to work in their workplaces, attend school, and function in society without suffering involuntary harm.

## 5 Conclusion

This study found that common fragranced products can trigger adverse effects throughout the Australian population, with consequences for public health, workplaces, businesses, and societal wellbeing. It also indicates that some relatively straightforward and inexpensive approaches, such as fragrance-free policies, could not only reduce health risks but also increase revenues and societal access. While research is needed to fully understand why fragranced products are associated with a range of adverse health effects, and in a substantial portion of the population, it is important to take steps in the meantime to reduce or eliminate exposure for prevention and public health.

## Conflicts of interest

None.

## Appendix A Supplementary data

Supplementary material 1. Image 1

Supplementary material 2. Image 2
