# Supplementary material for: Health and societal effects from exposure to fragranced consumer products
Source: Prev Med Rep. 2016 Nov 14;5:45–7. doi: 10.1016/j.pmedr.2016.11.011 (PMC5122698; doi:10.1016/j.pmedr.2016.11.011)
Supplement: Supplementary file 2 — Supplementary material 2. [file mmc2.pdf]

Australia Survey Results

Q1 Which fragranced products are you exposed to, at least once a week, from your own use? Air fresheners and deodorizers (e.g., sprays, solids, oils, disks)

		Frequency	Percent	Valid Percent	Cumulative Percent
Valid	No	365	33.2	33.2	33.2
	Yes	733	66.8	66.8	100.0
	Total	1098	100.0	100.0	

Q1 Which fragranced products are you exposed to, at least once a week, from your own use? Personal care products (e.g., soaps, hand sanitizer, lotions, deodorant, sunscreen, shampoos)

		Frequency	Percent	Valid Percent	Cumulative Percent
Valid	No	92	8.4	8.4	8.4
	Yes	1006	91.6	91.6	100.0
	Total	1098	100.0	100.0	

Q1 Which fragranced products are you exposed to, at least once a week, from your own use? Cleaning supplies (e.g., all-purpose cleaners, disinfectants, and dishwashing soap)

		Frequency	Percent	Valid Percent	Cumulative Percent
Valid	No	185	16.8	16.8	16.8
	Yes	913	83.2	83.2	100.0
	Total	1098	100.0	100.0	

Q1 Which fragranced products are you exposed to, at least once a week, from your own use? Laundry products (e.g., detergents, fabric softeners, dryer sheets)

		Frequency	Percent	Valid Percent	Cumulative Percent
Valid	No	172	15.7	15.7	15.7
	Yes	926	84.3	84.3	100.0
	Total	1098	100.0	100.0	

Q1 Which fragranced products are you exposed to, at least once a week, from your own use? Household products (e.g., scented candles, toilet paper, trash bags, baby products)

		Frequency	Percent	Valid Percent	Cumulative Percent
Valid	No	251	22.9	22.9	22.9
	Yes	847	77.1	77.1	100.0
	Total	1098	100.0	100.0	

Q1 Which fragranced products are you exposed to, at least once a week, from your own use? Fragrance (e.g., perfume, cologne, after-shave)

		Frequency	Percent	Valid Percent	Cumulative Percent
Valid	No	334	30.4	30.4	30.4
	Yes	764	69.6	69.6	100.0
	Total	1098	100.0	100.0	

Q1 Which fragranced products are you exposed to, at least once a week, from your own use? Other

		Frequency	Percent	Valid Percent	Cumulative Percent
Valid	No	1073	97.7	97.7	97.7
	Yes	25	2.3	2.3	100.0
	Total	1098	100.0	100.0	

Q1 Which fragranced products are you exposed to, at least once a week, from your own use? None

		Frequency	Percent	Valid Percent	Cumulative Percent
Valid	No	1076	98.0	98.0	98.0
	Yes	22	2.0	2.0	100.0
	Total	1098	100.0	100.0	

Q1a Which fragranced products are you exposed to, at least once a week, from others' use? Air fresheners and deodorizers (e.g., sprays, solids, oils, disks)

		Frequency	Percent	Valid Percent	Cumulative Percent
Valid	No	540	49.2	49.2	49.2
	Yes	558	50.8	50.8	100.0
	Total	1098	100.0	100.0	

Q1a Which fragranced products are you exposed to, at least once a week, from others' use? Personal care products (e.g., soaps, hand sanitizer, lotions, deodorant, sunscreen, shampoos)

		Frequency	Percent	Valid Percent	Cumulative Percent
Valid	No	423	38.5	38.5	38.5
	Yes	675	61.5	61.5	100.0
	Total	1098	100.0	100.0	

Q1a Which fragranced products are you exposed to, at least once a week, from others' use? Cleaning supplies (e.g., all-purpose cleaners, disinfectants, and dishwashing soap)

		Frequency	Percent	Valid Percent	Cumulative Percent
Valid	No	541	49.3	49.3	49.3
	Yes	557	50.7	50.7	100.0
	Total	1098	100.0	100.0	

Q1a Which fragranced products are you exposed to, at least once a week, from others' use? Laundry products (e.g., detergents, fabric softeners, dryer sheets)

		Frequency	Percent	Valid Percent	Cumulative Percent
Valid	No	612	55.7	55.7	55.7
	Yes	486	44.3	44.3	100.0
	Total	1098	100.0	100.0	

Q1a Which fragranced products are you exposed to, at least once a week, from others' use? Household products (e.g., scented candles, toilet paper, trash bags, baby products)

		Frequency	Percent	Valid Percent	Cumulative Percent
Valid	No	553	50.4	50.4	50.4
	Yes	545	49.6	49.6	100.0
	Total	1098	100.0	100.0	

Q1a Which fragranced products are you exposed to, at least once a week, from others' use? Fragrance (e.g., perfume, cologne, after-shave)

		Frequency	Percent	Valid Percent	Cumulative Percent
Valid	No	354	32.2	32.2	32.2
	Yes	744	67.8	67.8	100.0
	Total	1098	100.0	100.0	

Q1a Which fragranced products are you exposed to, at least once a week, from others' use? Other

		Frequency	Percent	Valid Percent	Cumulative Percent
Valid	No	1078	98.2	98.2	98.2
	Yes	20	1.8	1.8	100.0
	Total	1098	100.0	100.0	

Q1a Which fragranced products are you exposed to, at least once a week, from others' use? None

		Frequency	Percent	Valid Percent	Cumulative Percent
Valid	No	967	88.1	88.1	88.1
	Yes	131	11.9	11.9	100.0
	Total	1098	100.0	100.0	

Q2 Do you experience any health problems when exposed to air fresheners or deodorizers?

		Frequency	Percent	Valid Percent	Cumulative Percent
Valid	Yes	180	16.4	16.4	16.4
	No	760	69.2	69.2	85.6
	Don't know/not sure	157	14.3	14.3	99.9
	Decline to answer	1	.1	.1	100.0
	Total	1098	100.0	100.0	

BA Which of the following health problems do you experience when exposed to air fresheners or deodorizers? Migraine headaches

		Frequency	Percent	Valid Percent	Cumulative Percent
Valid	No	134	12.2	74.4	74.4
	Yes	46	4.2	25.6	100.0
	Total	180	16.4	100.0	
Missing	System	918	83.6		
Total		1098	100.0		

BA Which of the following health problems do you experience when exposed to air fresheners or deodorizers? Asthma attacks

		Frequency	Percent	Valid Percent	Cumulative Percent
Valid	No	131	11.9	72.8	72.8
	Yes	49	4.5	27.2	100.0
	Total	180	16.4	100.0	
Missing	System	918	83.6		
Total		1098	100.0		

BA Which of the following health problems do you experience when exposed to air fresheners or deodorizers? Neurological problems (e.g., dizziness, seizures, head pain, fainting, loss of coordination)

		Frequency	Percent	Valid Percent	Cumulative Percent
Valid	No	156	14.2	86.7	86.7
	Yes	24	2.2	13.3	100.0
	Total	180	16.4	100.0	
Missing	System	918	83.6		
Total		1098	100.0		

BA Which of the following health problems do you experience when exposed to air fresheners or deodorizers? Respiratory problems (e.g., difficulty breathing, coughing, shortness of breath)

		Frequency	Percent	Valid Percent	Cumulative Percent
Valid	No	80	7.3	44.4	44.4
	Yes	100	9.1	55.6	100.0
	Total	180	16.4	100.0	
Missing	System	918	83.6		
Total		1098	100.0		

BA Which of the following health problems do you experience when exposed to air fresheners or deodorizers? Skin problems (e.g., rashes, hives, red skin, tingling skin, dermatitis)

		Frequency	Percent	Valid Percent	Cumulative Percent
Valid	No	127	11.6	70.6	70.6
	Yes	53	4.8	29.4	100.0
	Total	180	16.4	100.0	
Missing	System	918	83.6		
Total		1098	100.0		

BA Which of the following health problems do you experience when exposed to air fresheners or deodorizers? Cognitive problems (e.g., difficulties thinking, concentrating, or remembering)

		Frequency	Percent	Valid Percent	Cumulative Percent
Valid	No	159	14.5	88.3	88.3
	Yes	21	1.9	11.7	100.0
	Total	180	16.4	100.0	
Missing	System	918	83.6		
Total		1098	100.0		

BA Which of the following health problems do you experience when exposed to air fresheners or deodorizers? Mucosal symptoms (e.g., watery or red eyes, nasal congestion, sneezing)

		Frequency	Percent	Valid Percent	Cumulative Percent
Valid	No	112	10.2	62.2	62.2
	Yes	68	6.2	37.8	100.0
	Total	180	16.4	100.0	
Missing	System	918	83.6		
Total		1098	100.0		

BA Which of the following health problems do you experience when exposed to air fresheners or deodorizers? Immune system problems (e.g., swollen lymph glands, fever, fatigue)

		Frequency	Percent	Valid Percent	Cumulative Percent
Valid	No	160	14.6	88.9	88.9
	Yes	20	1.8	11.1	100.0
	Total	180	16.4	100.0	
Missing	System	918	83.6		
Total		1098	100.0		

BA Which of the following health problems do you experience when exposed to air fresheners or deodorizers? Gastrointestinal problems (e.g., nausea, bloating, cramping, diarrhea)

		Frequency	Percent	Valid Percent	Cumulative Percent
Valid	No	164	14.9	91.1	91.1
	Yes	16	1.5	8.9	100.0
	Total	180	16.4	100.0	
Missing	System	918	83.6		
Total		1098	100.0		

BA Which of the following health problems do you experience when exposed to air fresheners or deodorizers? Cardiovascular problems (e.g., fast or irregular heartbeat, jitteriness, chest discomfort)

		Frequency	Percent	Valid Percent	Cumulative Percent
Valid	No	159	14.5	88.3	88.3
	Yes	21	1.9	11.7	100.0
	Total	180	16.4	100.0	
Missing	System	918	83.6		
Total		1098	100.0		

BA Which of the following health problems do you experience when exposed to air fresheners or deodorizers? Musculoskeletal problems (e.g., muscle or joint pain, cramps, weakness)

		Frequency	Percent	Valid Percent	Cumulative Percent
Valid	No	162	14.8	90.0	90.0
	Yes	18	1.6	10.0	100.0
	Total	180	16.4	100.0	
Missing	System	918	83.6		
Total		1098	100.0		

BA Which of the following health problems do you experience when exposed to air fresheners or deodorizers? Other

		Frequency	Percent	Valid Percent	Cumulative Percent
Valid	No	174	15.8	96.7	96.7
	Yes	6	.5	3.3	100.0
	Total	180	16.4	100.0	
Missing	System	918	83.6		
Total		1098	100.0		

Q3 Do you experience any health problems from the scent of laundry products coming from a dryer vent?

		Frequency	Percent	Valid Percent	Cumulative Percent
Valid	Yes	67	6.1	6.1	6.1
	No	909	82.8	82.8	88.9
	Don't know/not sure	120	10.9	10.9	99.8
	Decline to answer	2	.2	.2	100.0
	Total	1098	100.0	100.0	

BA Which of the following health problems do you suffer from the scent of laundry products coming from a dryer vent? Migraine headaches

		Frequency	Percent	Valid Percent	Cumulative Percent
Valid	No	53	4.8	79.1	79.1
	Yes	14	1.3	20.9	100.0
	Total	67	6.1	100.0	
Missing	System	1031	93.9		
Total		1098	100.0		

BA Which of the following health problems do you suffer from the scent of laundry products coming from a dryer vent? Asthma attacks

		Frequency	Percent	Valid Percent	Cumulative Percent
Valid	No	52	4.7	77.6	77.6
	Yes	15	1.4	22.4	100.0
	Total	67	6.1	100.0	
Missing	System	1031	93.9		
Total		1098	100.0		

BA Which of the following health problems do you suffer from the scent of laundry products coming from a dryer vent? Neurological problems (e.g., dizziness, seizures, head pain, fainting, loss of coordination)

		Frequency	Percent	Valid Percent	Cumulative Percent
Valid	No	59	5.4	88.1	88.1
	Yes	8	.7	11.9	100.0
	Total	67	6.1	100.0	
Missing	System	1031	93.9		
Total		1098	100.0		

BA Which of the following health problems do you suffer from the scent of laundry products coming from a dryer vent? Respiratory problems (e.g., difficulty breathing, coughing, shortness of breath)

		Frequency	Percent	Valid Percent	Cumulative Percent
Valid	No	39	3.6	58.2	58.2
	Yes	28	2.6	41.8	100.0
	Total	67	6.1	100.0	
Missing	System	1031	93.9		
Total		1098	100.0		

BA Which of the following health problems do you suffer from the scent of laundry products coming from a dryer vent? Skin problems (e.g., rashes, hives, red skin, tingling skin, dermatitis)

		Frequency	Percent	Valid Percent	Cumulative Percent
Valid	No	44	4.0	65.7	65.7
	Yes	23	2.1	34.3	100.0
	Total	67	6.1	100.0	
Missing	System	1031	93.9		
Total		1098	100.0		

BA Which of the following health problems do you suffer from the scent of laundry products coming from a dryer vent? Cognitive problems (e.g., difficulties thinking, concentrating, or remembering)

		Frequency	Percent	Valid Percent	Cumulative Percent
Valid	No	56	5.1	83.6	83.6
	Yes	11	1.0	16.4	100.0
	Total	67	6.1	100.0	
Missing	System	1031	93.9		
Total		1098	100.0		

BA Which of the following health problems do you suffer from the scent of laundry products coming from a dryer vent? Mucosal symptoms (e.g., watery or red eyes, nasal congestion, sneezing)

		Frequency	Percent	Valid Percent	Cumulative Percent
Valid	No	50	4.6	74.6	74.6
	Yes	17	1.5	25.4	100.0
	Total	67	6.1	100.0	
Missing	System	1031	93.9		
Total		1098	100.0		

BA Which of the following health problems do you suffer from the scent of laundry products coming from a dryer vent? Immune system problems (e.g., swollen lymph glands, fever, fatigue)

		Frequency	Percent	Valid Percent	Cumulative Percent
Valid	No	47	4.3	70.1	70.1
	Yes	20	1.8	29.9	100.0
	Total	67	6.1	100.0	
Missing	System	1031	93.9		
Total		1098	100.0		

BA Which of the following health problems do you suffer from the scent of laundry products coming from a dryer vent? Gastrointestinal problems (e.g., nausea, bloating, cramping, diarrhea)

		Frequency	Percent	Valid Percent	Cumulative Percent
Valid	No	54	4.9	80.6	80.6
	Yes	13	1.2	19.4	100.0
	Total	67	6.1	100.0	
Missing	System	1031	93.9		
Total		1098	100.0		

BA Which of the following health problems do you suffer from the scent of laundry products coming from a dryer vent? Cardiovascular problems (e.g., fast or irregular heartbeat, jitteriness, chest discomfort)

		Frequency	Percent	Valid Percent	Cumulative Percent
Valid	No	52	4.7	77.6	77.6
	Yes	15	1.4	22.4	100.0
	Total	67	6.1	100.0	
Missing	System	1031	93.9		
Total		1098	100.0		

BA Which of the following health problems do you suffer from the scent of laundry products coming from a dryer vent? Musculoskeletal problems (e.g., muscle or joint pain, cramps, weakness)

		Frequency	Percent	Valid Percent	Cumulative Percent
Valid	No	57	5.2	85.1	85.1
	Yes	10	.9	14.9	100.0
	Total	67	6.1	100.0	
Missing	System	1031	93.9		
Total		1098	100.0		

BA Which of the following health problems do you suffer from the scent of laundry products coming from a dryer vent? Other

		Frequency	Percent	Valid Percent	Cumulative Percent
Valid	No	65	5.9	97.0	97.0
	Yes	2	.2	3.0	100.0
	Total	67	6.1	100.0	
Missing	System	1031	93.9		
Total		1098	100.0		

Q4 Do you experience any health problems from being in a room after it has been cleaned with scented products?

		Frequency	Percent	Valid Percent	Cumulative Percent
Valid	Yes	168	15.3	15.3	15.3
	No	817	74.4	74.4	89.7
	Don't know/not sure	110	10.0	10.0	99.7
	Decline to answer	3	.3	.3	100.0
	Total	1098	100.0	100.0	

BA Which of the following health problems do you experience from being in a room after it has been cleaned with scented? Migraine headaches

		Frequency	Percent	Valid Percent	Cumulative Percent
Valid	No	130	11.8	77.4	77.4
	Yes	38	3.5	22.6	100.0
	Total	168	15.3	100.0	
Missing	System	930	84.7		
Total		1098	100.0		

BA Which of the following health problems do you experience from being in a room after it has been cleaned with scented? Asthma attacks

		Frequency	Percent	Valid Percent	Cumulative Percent
Valid	No	141	12.8	83.9	83.9
	Yes	27	2.5	16.1	100.0
	Total	168	15.3	100.0	
Missing	System	930	84.7		
Total		1098	100.0		

BA Which of the following health problems do you experience from being in a room after it has been cleaned with scented? Neurological problems (e.g., dizziness, seizures, head pain, fainting, loss of coordination)

		Frequency	Percent	Valid Percent	Cumulative Percent
Valid	No	151	13.8	89.9	89.9
	Yes	17	1.5	10.1	100.0
	Total	168	15.3	100.0	
Missing	System	930	84.7		
Total		1098	100.0		

BA Which of the following health problems do you experience from being in a room after it has been cleaned with scented? Respiratory problems (e.g., difficulty breathing, coughing, shortness of breath)

		Frequency	Percent	Valid Percent	Cumulative Percent
Valid	No	86	7.8	51.2	51.2
	Yes	82	7.5	48.8	100.0
	Total	168	15.3	100.0	
Missing	System	930	84.7		
Total		1098	100.0		

BA Which of the following health problems do you experience from being in a room after it has been cleaned with scented? Skin problems (e.g., rashes, hives, red skin, tingling skin, dermatitis)

		Frequency	Percent	Valid Percent	Cumulative Percent
Valid	No	137	12.5	81.5	81.5
	Yes	31	2.8	18.5	100.0
	Total	168	15.3	100.0	
Missing	System	930	84.7		
Total		1098	100.0		

BA Which of the following health problems do you experience from being in a room after it has been cleaned with scented? Cognitive problems (e.g., difficulties thinking, concentrating, or remembering)

		Frequency	Percent	Valid Percent	Cumulative Percent
Valid	No	148	13.5	88.1	88.1
	Yes	20	1.8	11.9	100.0
	Total	168	15.3	100.0	
Missing	System	930	84.7		
Total		1098	100.0		

BA Which of the following health problems do you experience from being in a room after it has been cleaned with scented? Mucosal symptoms (e.g., watery or red eyes, nasal congestion, sneezing)

		Frequency	Percent	Valid Percent	Cumulative Percent
Valid	No	102	9.3	60.7	60.7
	Yes	66	6.0	39.3	100.0
	Total	168	15.3	100.0	
Missing	System	930	84.7		
Total		1098	100.0		

BA Which of the following health problems do you experience from being in a room after it has been cleaned with scented? Immune system problems (e.g., swollen lymph glands, fever, fatigue)

		Frequency	Percent	Valid Percent	Cumulative Percent
Valid	No	150	13.7	89.3	89.3
	Yes	18	1.6	10.7	100.0
	Total	168	15.3	100.0	
Missing	System	930	84.7		
Total		1098	100.0		

BA Which of the following health problems do you experience from being in a room after it has been cleaned with scented? Gastrointestinal problems (e.g., nausea, bloating, cramping, diarrhea)

		Frequency	Percent	Valid Percent	Cumulative Percent
Valid	No	152	13.8	90.5	90.5
	Yes	16	1.5	9.5	100.0
	Total	168	15.3	100.0	
Missing	System	930	84.7		
Total		1098	100.0		

BA Which of the following health problems do you experience from being in a room after it has been cleaned with scented? Cardiovascular problems (e.g., fast or irregular heartbeat, jitteriness, chest discomfort)

		Frequency	Percent	Valid Percent	Cumulative Percent
Valid	No	154	14.0	91.7	91.7
	Yes	14	1.3	8.3	100.0
	Total	168	15.3	100.0	
Missing	System	930	84.7		
Total		1098	100.0		

BA Which of the following health problems do you experience from being in a room after it has been cleaned with scented? Musculoskeletal problems (e.g., muscle or joint pain, cramps, weakness)

		Frequency	Percent	Valid Percent	Cumulative Percent
Valid	No	157	14.3	93.5	93.5
	Yes	11	1.0	6.5	100.0
	Total	168	15.3	100.0	
Missing	System	930	84.7		
Total		1098	100.0		

BA Which of the following health problems do you experience from being in a room after it has been cleaned with scented? Other

		Frequency	Percent	Valid Percent	Cumulative Percent
Valid	No	161	14.7	95.8	95.8
	Yes	7	.6	4.2	100.0
	Total	168	15.3	100.0	
Missing	System	930	84.7		
Total		1098	100.0		

Q5 Do you experience any health problems from being near someone who is wearing a fragranced product?

		Frequency	Percent	Valid Percent	Cumulative Percent
Valid	Yes	213	19.4	19.4	19.4
	No	793	72.2	72.2	91.6
	Don't know/not sure	90	8.2	8.2	99.8
	Decline to answer	2	.2	.2	100.0
	Total	1098	100.0	100.0	

BA Which of the following health problems do you experience from being near someone who is wearing a fragranced product? Migraine headaches

		Frequency	Percent	Valid Percent	Cumulative Percent
Valid	No	159	14.5	74.6	74.6
	Yes	54	4.9	25.4	100.0
	Total	213	19.4	100.0	
Missing	System	885	80.6		
Total		1098	100.0		

BA Which of the following health problems do you experience from being near someone who is wearing a fragranced product? Asthma attacks

		Frequency	Percent	Valid Percent	Cumulative Percent
Valid	No	176	16.0	82.6	82.6
	Yes	37	3.4	17.4	100.0
	Total	213	19.4	100.0	
Missing	System	885	80.6		
Total		1098	100.0		

BA Which of the following health problems do you experience from being near someone who is wearing a fragranced product? Neurological problems (e.g., dizziness, seizures, head pain, fainting, loss of coordination)

		Frequency	Percent	Valid Percent	Cumulative Percent
Valid	No	187	17.0	87.8	87.8
	Yes	26	2.4	12.2	100.0
	Total	213	19.4	100.0	
Missing	System	885	80.6		
Total		1098	100.0		

BA Which of the following health problems do you experience from being near someone who is wearing a fragranced product? Respiratory problems (e.g., difficulty breathing, coughing, shortness of breath)

		Frequency	Percent	Valid Percent	Cumulative Percent
Valid	No	122	11.1	57.3	57.3
	Yes	91	8.3	42.7	100.0
	Total	213	19.4	100.0	
Missing	System	885	80.6		
Total		1098	100.0		

BA Which of the following health problems do you experience from being near someone who is wearing a fragranced product? Skin problems (e.g., rashes, hives, red skin, tingling skin, dermatitis)

		Frequency	Percent	Valid Percent	Cumulative Percent
Valid	No	194	17.7	91.1	91.1
	Yes	19	1.7	8.9	100.0
	Total	213	19.4	100.0	
Missing	System	885	80.6		
Total		1098	100.0		

BA Which of the following health problems do you experience from being near someone who is wearing a fragranced product? Cognitive problems (e.g., difficulties thinking, concentrating, or remembering)

		Frequency	Percent	Valid Percent	Cumulative Percent
Valid	No	196	17.9	92.0	92.0
	Yes	17	1.5	8.0	100.0
	Total	213	19.4	100.0	
Missing	System	885	80.6		
Total		1098	100.0		

BA Which of the following health problems do you experience from being near someone who is wearing a fragranced product? Mucosal symptoms (e.g., watery or red eyes, nasal congestion, sneezing)

		Frequency	Percent	Valid Percent	Cumulative Percent
Valid	No	126	11.5	59.2	59.2
	Yes	87	7.9	40.8	100.0
	Total	213	19.4	100.0	
Missing	System	885	80.6		
Total		1098	100.0		

BA Which of the following health problems do you experience from being near someone who is wearing a fragranced product? Immune system problems (e.g., swollen lymph glands, fever, fatigue)

		Frequency	Percent	Valid Percent	Cumulative Percent
Valid	No	199	18.1	93.4	93.4
	Yes	14	1.3	6.6	100.0
	Total	213	19.4	100.0	
Missing	System	885	80.6		
Total		1098	100.0		

BA Which of the following health problems do you experience from being near someone who is wearing a fragranced product? Gastrointestinal problems (e.g., nausea, bloating, cramping, diarrhea)

		Frequency	Percent	Valid Percent	Cumulative Percent
Valid	No	197	17.9	92.5	92.5
	Yes	16	1.5	7.5	100.0
	Total	213	19.4	100.0	
Missing	System	885	80.6		
Total		1098	100.0		

BA Which of the following health problems do you experience from being near someone who is wearing a fragranced product? Cardiovascular problems (e.g., fast or irregular heartbeat, jitteriness, chest discomfort)

		Frequency	Percent	Valid Percent	Cumulative Percent
Valid	No	200	18.2	93.9	93.9
	Yes	13	1.2	6.1	100.0
	Total	213	19.4	100.0	
Missing	System	885	80.6		
Total		1098	100.0		

BA Which of the following health problems do you experience from being near someone who is wearing a fragranced product? Musculoskeletal problems (e.g., muscle or joint pain, cramps, weakness)

		Frequency	Percent	Valid Percent	Cumulative Percent
Valid	No	200	18.2	93.9	93.9
	Yes	13	1.2	6.1	100.0
	Total	213	19.4	100.0	
Missing	System	885	80.6		
Total		1098	100.0		

BA Which of the following health problems do you experience from being near someone who is wearing a fragranced product? Other

		Frequency	Percent	Valid Percent	Cumulative Percent
Valid	No	204	18.6	95.8	95.8
	Yes	9	.8	4.2	100.0
	Total	213	19.4	100.0	
Missing	System	885	80.6		
Total		1098	100.0		

Q6 In general, do you experience any health problems from exposure to any type of fragranced product?

		Frequency	Percent	Valid Percent	Cumulative Percent
Valid	Yes	223	20.3	20.3	20.3
	No	778	70.9	70.9	91.2
	Don't know/not sure	96	8.7	8.7	99.9
	Decline to answer	1	.1	.1	100.0
	Total	1098	100.0	100.0	

BA Which of the following health problems do you experience from exposure to any type of fragranced product? Migraine headaches

		Frequency	Percent	Valid Percent	Cumulative Percent
Valid	No	172	15.7	77.1	77.1
	Yes	51	4.6	22.9	100.0
	Total	223	20.3	100.0	
Missing	System	875	79.7		
Total		1098	100.0		

BA Which of the following health problems do you experience from exposure to any type of fragranced product? Asthma attacks

		Frequency	Percent	Valid Percent	Cumulative Percent
Valid	No	183	16.7	82.1	82.1
	Yes	40	3.6	17.9	100.0
	Total	223	20.3	100.0	
Missing	System	875	79.7		
Total		1098	100.0		

BA Which of the following health problems do you experience from exposure to any type of fragranced product? Neurological problems (e.g., dizziness, seizures, head pain, fainting, loss of coordination)

		Frequency	Percent	Valid Percent	Cumulative Percent
Valid	No	201	18.3	90.1	90.1
	Yes	22	2.0	9.9	100.0
	Total	223	20.3	100.0	
Missing	System	875	79.7		
Total		1098	100.0		

BA Which of the following health problems do you experience from exposure to any type of fragranced product? Respiratory problems (e.g., difficulty breathing, coughing, shortness of breath)

		Frequency	Percent	Valid Percent	Cumulative Percent
Valid	No	113	10.3	50.7	50.7
	Yes	110	10.0	49.3	100.0
	Total	223	20.3	100.0	
Missing	System	875	79.7		
Total		1098	100.0		

BA Which of the following health problems do you experience from exposure to any type of fragranced product? Skin problems (e.g., rashes, hives, red skin, tingling skin, dermatitis)

		Frequency	Percent	Valid Percent	Cumulative Percent
Valid	No	169	15.4	75.8	75.8
	Yes	54	4.9	24.2	100.0
	Total	223	20.3	100.0	
Missing	System	875	79.7		
Total		1098	100.0		

BA Which of the following health problems do you experience from exposure to any type of fragranced product? Cognitive problems (e.g., difficulties thinking, concentrating, or remembering)

		Frequency	Percent	Valid Percent	Cumulative Percent
Valid	No	204	18.6	91.5	91.5
	Yes	19	1.7	8.5	100.0
	Total	223	20.3	100.0	
Missing	System	875	79.7		
Total		1098	100.0		

BA Which of the following health problems do you experience from exposure to any type of fragranced product? Mucosal symptoms (e.g., watery or red eyes, nasal congestion, sneezing)

		Frequency	Percent	Valid Percent	Cumulative Percent
Valid	No	140	12.8	62.8	62.8
	Yes	83	7.6	37.2	100.0
	Total	223	20.3	100.0	
Missing	System	875	79.7		
Total		1098	100.0		

BA Which of the following health problems do you experience from exposure to any type of fragranced product? Immune system problems (e.g., swollen lymph glands, fever, fatigue)

		Frequency	Percent	Valid Percent	Cumulative Percent
Valid	No	210	19.1	94.2	94.2
	Yes	13	1.2	5.8	100.0
	Total	223	20.3	100.0	
Missing	System	875	79.7		
Total		1098	100.0		

BA Which of the following health problems do you experience from exposure to any type of fragranced product? Gastrointestinal problems (e.g., nausea, bloating, cramping, diarrhea)

		Frequency	Percent	Valid Percent	Cumulative Percent
Valid	No	209	19.0	93.7	93.7
	Yes	14	1.3	6.3	100.0
	Total	223	20.3	100.0	
Missing	System	875	79.7		
Total		1098	100.0		

BA Which of the following health problems do you experience from exposure to any type of fragranced product? Cardiovascular problems (e.g., fast or irregular heartbeat, jitteriness, chest discomfort)

		Frequency	Percent	Valid Percent	Cumulative Percent
Valid	No	210	19.1	94.2	94.2
	Yes	13	1.2	5.8	100.0
	Total	223	20.3	100.0	
Missing	System	875	79.7		
Total		1098	100.0		

BA Which of the following health problems do you experience from exposure to any type of fragranced product? Musculoskeletal problems (e.g., muscle or joint pain, cramps, weakness)

		Frequency	Percent	Valid Percent	Cumulative Percent
Valid	No	212	19.3	95.1	95.1
	Yes	11	1.0	4.9	100.0
	Total	223	20.3	100.0	
Missing	System	875	79.7		
Total		1098	100.0		

BA Which of the following health problems do you experience from exposure to any type of fragranced product? Other

		Frequency	Percent	Valid Percent	Cumulative Percent
Valid	No	215	19.6	96.4	96.4
	Yes	8	.7	3.6	100.0
	Total	223	20.3	100.0	
Missing	System	875	79.7		
Total		1098	100.0		

**Product Use**	own use	others' use
Total	1098	1098
	100.00%	100.00%
Yes (Net)	1076	967
	98.00%	88.10%
Air fresheners and deodorizers	733	558
	66.80%	50.80%
Personal care products	1006	675
	91.60%	61.50%
Cleaning supplies	913	557
	83.20%	50.70%
Laundry products	926	486
	84.30%	44.30%
Household products	847	545
	77.10%	49.60%
Fragrance	764	744
	69.60%	67.80%
Other	25	20
	2.30%	1.80%
None	22	131
	2.00%	11.90%
Total (own use, others' use, or both) = 98.5%		

**Health Effects from Product Exposure**	
	1098
	100.00%
Migraine headaches	110
	10.00%
Asthma attacks	83
	7.60%
Neurological problems	49
	4.50%
Respiratory problems	183
	16.70%
Skin problems	104
	9.50%
Cognitive problems	45
	4.10%
Mucosal symptoms	154
	14.00%
Immune system problems	36
	3.30%
Gastrointestinal problems	36
	3.30%
Cardiovascular problems	33
	3.00%
Musculoskeletal problems	29
	2.60%
Other	21
	1.90%
Total (reporting one or more health effects) = 33.0%	

B2 Do any of these health problems mean a total or partial loss of bodily or mental functions, for you personally?

		Frequency	Percent	Valid Percent	Cumulative Percent
Valid	Yes	62	5.6	17.1	17.1
	No	269	24.5	74.3	91.4
	Don't know/not sure	31	2.8	8.6	100.0
	Total	362	33.0	100.0	
Missing	System	736	67.0		
Total		1098	100.0		

Q11 Have you ever been unable or reluctant to use the toilets in a public place, because of the presence of an air freshener, deodorizer, or scented product?

		Frequency	Percent	Valid Percent	Cumulative Percent
Valid	Yes	127	11.6	11.6	11.6
	No	920	83.8	83.8	95.4
	Neutral/not sure	49	4.5	4.5	99.8
	Decline to answer	2	.2	.2	100.0
	Total	1098	100.0	100.0	

Q12 If you enter a business, and you smell air fresheners or some fragranced product, do you want to leave as quickly as possible?

		Frequency	Percent	Valid Percent	Cumulative Percent
Valid	Yes	183	16.7	16.7	16.7
	No	773	70.4	70.4	87.1
	Neutral/not sure	141	12.8	12.8	99.9
	Decline to answer	1	.1	.1	100.0
	Total	1098	100.0	100.0	

Q14 Have you ever been unable or reluctant to wash your hands with soap in a public place, because you know or suspect that the soap is fragranced?

		Frequency	Percent	Valid Percent	Cumulative Percent
Valid	Yes	113	10.3	10.3	10.3
	No	911	83.0	83.0	93.3
	Neutral/not sure	72	6.6	6.6	99.8
	Decline to answer	2	.2	.2	100.0
	Total	1098	100.0	100.0	

Q15 Are you aware that a 'fragrance' in a product is typically a chemical mixture of several dozen to several hundred chemicals, mostly synthetic and derived from petrochemicals?

		Frequency	Percent	Valid Percent	Cumulative Percent
Valid	Yes	446	40.6	40.6	40.6
	No	518	47.2	47.2	87.8
	Don't know/not sure	132	12.0	12.0	99.8
	Decline to answer	2	.2	.2	100.0
	Total	1098	100.0	100.0	

Q16 Are you aware that fragrance chemicals do not need to be fully disclosed on the product label or material safety data sheet?

		Frequency	Percent	Valid Percent	Cumulative Percent
Valid	Yes	197	17.9	17.9	17.9
	No	753	68.6	68.6	86.5
	Don't know/not sure	145	13.2	13.2	99.7
	Decline to answer	3	.3	.3	100.0
	Total	1098	100.0	100.0	

Q17 Are you aware that fragranced products typically emit hazardous air pollutants such as formaldehyde?

		Frequency	Percent	Valid Percent	Cumulative Percent
Valid	Yes	203	18.5	18.5	18.5
	No	757	68.9	68.9	87.4
	Don't know/not sure	134	12.2	12.2	99.6
	Decline to answer	4	.4	.4	100.0
	Total	1098	100.0	100.0	

Q18 Are you aware that even so-called natural, green, and organic fragranced products typically emit hazardous air pollutants?

		Frequency	Percent	Valid Percent	Cumulative Percent
Valid	Yes	164	14.9	14.9	14.9
	No	809	73.7	73.7	88.6
	Don't know/not sure	121	11.0	11.0	99.6
	Decline to answer	4	.4	.4	100.0
	Total	1098	100.0	100.0	

Q19a If you knew that a fragranced product emitted hazardous air pollutants, would you still use it?

		Frequency	Percent	Valid Percent	Cumulative Percent
Valid	Yes	144	13.1	13.1	13.1
	No	618	56.3	56.3	69.4
	Don't know/not sure	334	30.4	30.4	99.8
	Decline to answer	2	.2	.2	100.0
	Total	1098	100.0	100.0	

Q19b Have you ever been prevented from going to some place because you would be exposed to a fragrance product that would make you sick?

		Frequency	Percent	Valid Percent	Cumulative Percent
Valid	Yes	165	15.0	15.0	15.0
	No	832	75.8	75.8	90.8
	Don't know/not sure	99	9.0	9.0	99.8
	Decline to answer	2	.2	.2	100.0
	Total	1098	100.0	100.0	

Q19 Has any exposure to fragranced products in your work environment caused you to become sick, lose work days, or lose a job?

		Frequency	Percent	Valid Percent	Cumulative Percent
Valid	Yes	85	7.7	7.7	7.7
	No	929	84.6	84.6	92.3
	Don't know/not sure	81	7.4	7.4	99.7
	Decline to answer	3	.3	.3	100.0
	Total	1098	100.0	100.0	

Q22 Would you be supportive of a fragrance-free policy in the workplace?

		Frequency	Percent	Valid Percent	Cumulative Percent
Valid	Yes	470	42.8	42.8	42.8
	No	244	22.2	22.2	65.0
	Neutral/not sure	381	34.7	34.7	99.7
	Decline to answer	3	.3	.3	100.0
	Total	1098	100.0	100.0	

Q24 Would you prefer that health care facilities and health care professionals be fragrance-free?

		Frequency	Percent	Valid Percent	Cumulative Percent
Valid	Yes	474	43.2	43.2	43.2
	No	277	25.2	25.2	68.4
	Neutral/not sure	342	31.1	31.1	99.5
	Decline to answer	5	.5	.5	100.0
	Total	1098	100.0	100.0	

Q25 If you had a choice between ... flying on an airplane that pumped scented air throughout the passenger cabin, OR flying on an airplane that did not pump scented air throughout the passenger cabin. Which would you choose?

		Frequency	Percent	Valid Percent	Cumulative Percent
Valid	Airplane with scented air	179	16.3	16.3	16.3
	Airplane without scented air	634	57.7	57.7	74.0
	Neutral/not sure	283	25.8	25.8	99.8
	Decline to answer	2	.2	.2	100.0
	Total	1098	100.0	100.0	

Q26 If you had a choice between... staying in a hotel with fragranced air OR staying in a hotel without fragranced air. Which would you choose?

		Frequency	Percent	Valid Percent	Cumulative Percent
Valid	Hotel with fragranced air	249	22.7	22.7	22.7
	Hotel without fragranced air	610	55.6	55.6	78.2
	Neutral/not sure	237	21.6	21.6	99.8
	Decline to answer	2	.2	.2	100.0
	Total	1098	100.0	100.0	

Q27 What is your household annual income?

		Frequency	Percent	Valid Percent	Cumulative Percent
Valid	Less than $10,000	42	3.8	3.8	3.8
	$10,000 - $49,999	237	21.6	21.6	25.4
	$50,000 - $99,999	375	34.2	34.2	59.6
	$100,000-$149,999	206	18.8	18.8	78.3
	$150,000-$200,000	81	7.4	7.4	85.7
	Over $200,000	37	3.4	3.4	89.1
	Decline to answer	120	10.9	10.9	100.0
	Total	1098	100.0	100.0	

Region

		Frequency	Percent	Valid Percent	Cumulative Percent
Valid	Australian Capital Territory	20	1.8	1.8	1.8
	New South Wales	360	32.8	32.8	34.6
	Northern Territory	7	.6	.6	35.2
	Queensland	217	19.8	19.8	55.0
	South Australia	85	7.7	7.7	62.8
	Tasmania	25	2.3	2.3	65.0
	Victoria	278	25.3	25.3	90.3
	Western Australia	106	9.7	9.7	100.0
	Total	1098	100.0	100.0	

Gender

		Frequency	Percent	Valid Percent	Cumulative Percent
Valid	Male	543	49.5	49.5	49.5
	Female	555	50.5	50.5	100.0
	Total	1098	100.0	100.0	

Age

		Frequency	Percent	Valid Percent	Cumulative Percent
Valid	18-24	156	14.2	14.2	14.2
	25-34	239	21.8	21.8	36.0
	35-44	256	23.3	23.3	59.3
	45-54	241	21.9	21.9	81.2
	55-65	206	18.8	18.8	100.0
	Total	1098	100.0	100.0	

		Frequency	Percent	Valid Percent	Cumulative Percent
Valid	AUS	1098	100.0	100.0	100.0
